# Dry Carbonate Sorbents for CO_2_ Capture from Flue Gases: Role of Support in Adsorption Efficiency and Thermal Stability

**DOI:** 10.3390/molecules30132859

**Published:** 2025-07-04

**Authors:** Bolatbek Khussain, Alexandr Sass, Alexandr Brodskiy, Murat Zhurinov, Ivan Torlopov, Kenzhegul Rakhmetova, Daulet Zhumadullaev, Yerzhan Boleubayev, Atabek Khussain, Abzal Kenessary, Adel Sarsenova, Tumen Darzhokov

**Affiliations:** 1 D.V. Sokolsky Institute of Fuel, Catalysis and Electrochemistry, 142, Kunaev Str., Almaty 050010, Kazakhstan; bolatbekh@mail.ru (B.K.); m.zhurinov@ifce.kz (M.Z.); myndfrea@gmail.com (I.T.); rahmetova_75@mail.ru (K.R.); dauletmmm@mail.ru (D.Z.); erzhan_bol@mail.ru (Y.B.); atabek.khussain@gmail.com (A.K.); 2 Kazakh Institute of Oil and Gas, 506/99, Seifullin Ave., Almaty 050000, Kazakhstan; a.kenessary@king.kz (A.K.); a.sarsenova@kbtu.kz (A.S.); darzhokov7@gmail.com (T.D.)

**Keywords:** CO_2_ capture, chemisorption, carbonate-based sorbents, kaolin, calcium carbonate, thermal stability

## Abstract

This study presents the results of an investigation of carbonate-containing sorbents for CO_2_ capture with natural support materials—kaolin and calcium carbonate—at various loadings of the active phase of Na_2_CO_3_. The effects of the support type on the distribution of the active component, phase composition, and pore structure of the sorbents were studied. It was found that a Na_2_CO_3_ loading of 25 wt.% provides the best balance between sorption capacity and technological feasibility. The thermal stability and regeneration capacity of the sorbents were evaluated under high-temperature conditions, revealing high thermal stability of the Na_2_CO_3_/CaCO_3_ system up to 1000 °C, along with its durability over multiple adsorption–desorption cycles. Kinetic studies on the Na_2_CO_3_/CaCO_3_ sorbent using the shrinking core model demonstrated that the overall CO_2_ chemisorption process is controlled by surface chemical reaction at temperatures below 50 °C. The obtained results demonstrate the high potential of CaCO_3_-based sorbents for practical applications in low-temperature CO_2_ capture technologies. A promising direction for the use of such sorbents within CCUS is the development of integrated systems, where CO_2_ capture is combined with its conversion into valuable products (e.g., methane, methanol, formic acid) through catalytic processes.

## 1. Introduction

Carbon dioxide (CO_2_) emissions are a major byproduct of energy production systems that rely on fossil fuels such as coal and hydrocarbons. CO_2_ is the primary contributor to the greenhouse effect, accounting for over 75% of all anthropogenic greenhouse gases [1], which has led many countries to implement emission quotas [2,3,4,5]. These global regulations have driven the development and implementation of carbon capture, utilization, and storage (CCUS) technologies, which aim to capture CO_2_ from flue gases emitted by power plants, industrial facilities, and other sources, followed by its transport for further utilization in various industries or long-term storage in geological formations, such as depleted oil and gas reservoirs [6,7,8]. Currently, CCUS technologies have been deployed in many forms in the United States, China, and several European countries, with the majority of projects focusing on geological storage due to the large volumes of CO_2_ produced, which exceed the current capacity for industrial utilization [7,8].

Efficient CO_2_ capture is a critical step for the implementation of CCS processes that also plays an important role in CCU schemes, especially when integrated with capture materials. Currently, more than 80% of existing CO_2_ capture facilities utilize amine-based technology, based on the chemical absorption of CO_2_ with the use of ethanolamine solutions. Despite the maturity and high efficiency of this method, its significant drawbacks—including high energy consumption for solvent regeneration, corrosivity, toxicity, and solvent degradation—drive the search for alternative CO_2_ capture approaches that can be scaled to industrial volumes [7,8,9].

Solid adsorbents represent one of the most promising alternatives to amine absorption. These materials offer low energy consumption, simple apparatus design, and operational flexibility, making them suitable for CO_2_ capture across a wide range of flue gas temperatures and compositions [8,9,10,11,12]. However, the development of effective adsorbents remains challenging due to the numerous performance requirements they must meet, including high sorption capacity, CO_2_ selectivity, low energy demand for desorption, thermal stability, mechanical strength, and scalability [11,12].

Among the most promising adsorption technologies for CO_2_ capture is the dry carbonate process (DCP), based on the reaction of CO_2_ with carbonate-based sorbents of the type Me_2_CO_3_/support (Me = Na, K) at 50–80 °C, followed by desorption at 100–150 °C [13,14,15,16]. This process is particularly well-suited for post-combustion CO_2_ capture from the flue gases of power plants fueled by solid fuels or natural gas due to the very large flow rates involved and their comparatively low temperatures.

Numerous formulations of carbonate-based sorbents have been reported in the literature, primarily using supports such as alumina, aluminosilicates, or activated carbon [17,18,19,20]. However, the potential of alternative supports, as well as their interaction with the active phase in such systems, remains insufficiently studied, despite their critical influence on the sorption efficiency and the mechanical properties of the materials [10,12].

Importantly, in addition to their use in conventional DCP, carbonate sorbents are also being explored as the basis for effective sorbent–catalyst systems. Given the basic nature of the carbonate phase, it is capable not only of capturing CO_2_ but also potentially facilitating its transformation to methane, methanol, and other valuable products within a single reaction cycle [9,11].

In this context, the present study focuses on the synthesis and characterization of carbonate-based sorbents supported on two distinct natural materials—kaolin and calcium carbonate (CaCO_3_)—selected due to their different physicochemical properties, which are expected to influence their behavior as CO_2_ sorbent supports.

## 2. Results

### 2.1. Physicochemical Characterization of Sorbent Samples

The synthesized sorbent samples, based on natural aluminosilicate (kaolin, Al_2_Si_2_O_5_(OH)_4_) and calcium carbonate (CaCO_3_), corresponding to the KS and LS series, respectively, were comprehensively characterized using a range of physicochemical techniques. The primary focus was on determining the phase and elemental composition of the samples and investigating their changes as a function of the active component loading (Na_2_CO_3_) and thermal treatment at elevated temperatures.

**Figure 1:**
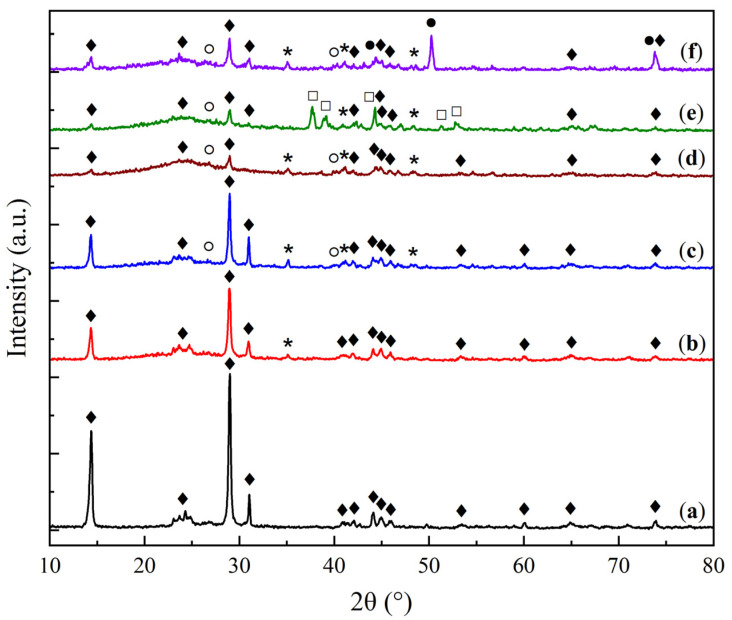
XRD patterns of kaolin (**a**) and sorbent samples: (**b**) KS10, (**c**) KS15, (**d**) KS25, (**e**) KS50, and (**f**) KS25M. (♦—kaolinite Al_2_Si_2_O_5_(OH)_4_, *—nahcolite NaHCO_3_, ○—natrite Na_2_CO_3_, □—thermonatrite Na_2_CO_3_·H_2_O, ●—periclase MgO.)

**Figure 2:**
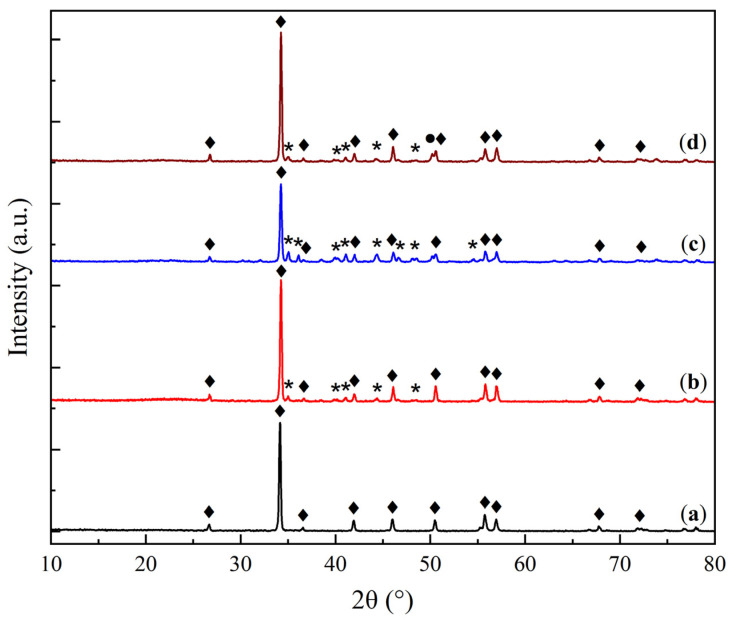
XRD patterns of CaCO_3_ (**a**) and sorbent samples: (**b**) LS25, (**c**) LS50, and (**d**) LS25M. (♦—calcite CaCO_3_, *—natrite Na_2_CO_3_, ●—periclase MgO.)

The X-ray diffraction (XRD) patterns of the KS and LS series samples are presented in Figure 1 and Figure 2, respectively, providing insights into the crystalline phases present in the sorbents and their transformation under varying conditions of Na_2_CO_3_ loading and thermal exposure.

The X-ray diffraction (XRD) analysis of the KS series samples (Figure 1) revealed the presence of a hydrated aluminosilicate phase corresponding to kaolinite (Al_2_Si_2_O_5_(OH)_4_, JCPDS 29-1488 [21]). In all Na_2_CO_3_-containing samples, regardless of the active component loading, a dispersed phase of sodium bicarbonate (NaHCO_3_) was detected (JCPDS 15-700 [22]). As the Na_2_CO_3_ loading increased, weak reflections corresponding to sodium carbonate (JCPDS 19-1130 [22]) were observed, and in the KS50 sample, additional peaks of thermonatrite (Na_2_CO_3_·H_2_O, JCPDS 8-448 [23]) were detected. The presence of this phase is likely due to local supersaturation of carbonate species during molding and drying, resulting from an excess of the active component. The introduction of magnesia (MgO) led to the appearance of strong reflections of the periclase phase (MgO, JCPDS 45–946 [24]).

With an increasing Na_2_CO_3_ loading in the KS series, a gradual decrease in the intensity of kaolinite reflections was observed, accompanied by an increase in the intensity of NaHCO_3_ peaks. This effect is attributed to the reduced fraction of the support, as well as the surface coverage of the support by a growing carbonate layer, as confirmed by SEM data.

In the LS series (Figure 2), the initial support is represented by calcite (CaCO_3_, JCPDS 37-1497 [25]). The XRD patterns of the LS sorbents show reflections of both calcite and natrite (Na_2_CO_3_). The LS25M sample demonstrated a weak reflection of the MgO phase, indicating its higher dispersion compared to KS25M.

As the active component loading increases in the LS series, the intensity of the calcite reflections decreases, while the intensity of the Na_2_CO_3_ peaks shows a slight increase. The relatively low intensity of carbonate phase peaks in both series is attributed to the high dispersion and amorphization of the active component on the support surface, as supported by the SEM data.

Crystallite size calculations using the Scherrer equation indicated that for the KS series, the kaolinite phase exhibits a crystallite size of 30–40 nm, while NaHCO_3_ shows a size range of 40–60 nm. As the Na_2_CO_3_ loading grows, the crystallite size of the support decreases, whereas the active component size increases (up to 30 and 60 nm for these two phases of the KS50 sample, respectively). A similar but less pronounced trend is observed for the LS series, where the calcite crystallite size is 55–60 nm, reaching a minimum of 55 nm for LS50, while the size of the active component ranges from 35 to 45 nm, increasing to 44 nm at the highest Na_2_CO_3_ loading. These results suggest that the crystallite sizes of both the support and the active phase remain relatively stable, with the active phase tending to form a fine, highly dispersed layer on the support surface.

**Figure 3:**
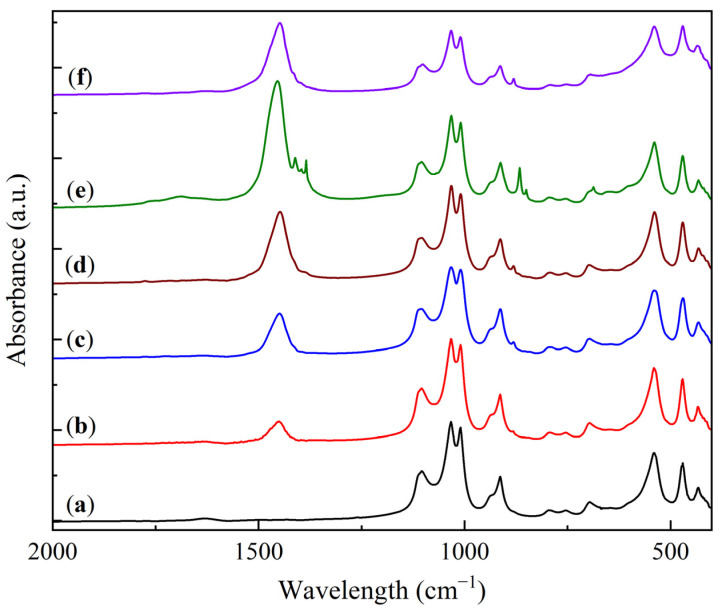
FTIR spectra of kaolinite (**a**) and sorbent samples: (**b**) KS10, (**c**) KS15, (**d**) KS25, (**e**) KS50, and (**f**) KS25M.

**Figure 4:**
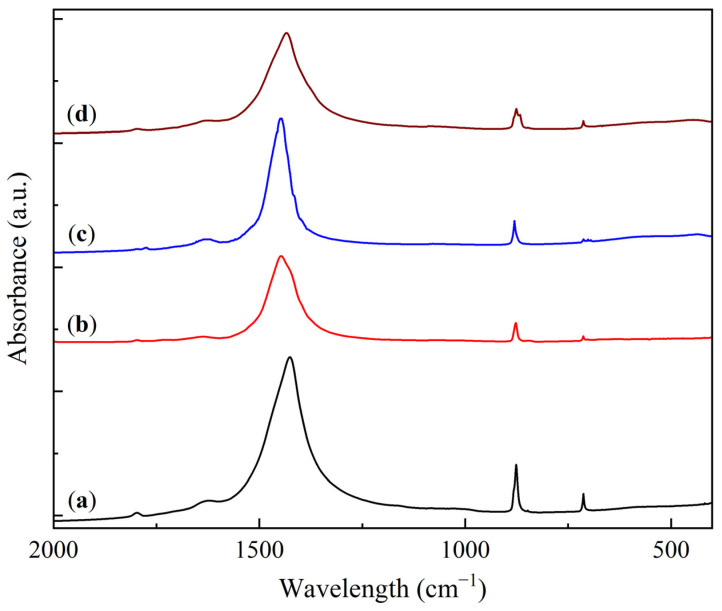
FTIR spectra of CaCO_3_ (**a**) and sorbent samples: (**b**) LS25, (**c**) LS50, and (**d**) LS25M.

The FTIR analysis results of the KS and LS series samples are presented in Figure 3 and Figure 4.

The FTIR spectra of the kaolin and the KS series samples (Figure 3) exhibit characteristic absorption bands in the range of 1100–400 cm^−1^ (1105, 1032, 1009, 913, 795, 753, 696, 540, 470, and 433 cm^−1^), which are typical of kaolinite [26,27]. The shape and position of the bands remain unchanged with increasing Na_2_CO_3_ loading, indicating the absence of significant interaction between the active component and the support.

In addition to the kaolinite bands, the FTIR spectra of the KS samples show absorption bands corresponding to carbonate and bicarbonate groups (1630, 1445, 1390, and 881 cm^−1^) [28,29]. The intensity of the broad band at 1445 cm^−1^ increases with the active component loading, reflecting the growing carbonate phase. In the KS50 sample, additional bands are observed at 1411, 1384, 866, 850, and 687 cm^−1^, also corresponding to carbonate (CO_3_^2−^) vibrations. These bands are attributed to the high concentration of carbonate-containing phases in this sample. The introduction of magnesia (MgO) results in the appearance of a characteristic broad absorption band in the region of 500–400 cm^−1^ [30]. This observation confirms the presence of a finely dispersed MgO phase in the modified samples.

The FTIR spectra of the initial CaCO_3_ and the LS series samples display characteristic carbonate absorption bands at 1799, 1627, 1427, 876, and 713 cm^−1^. Upon the addition of Na_2_CO_3_, the primary carbonate band (1425–1450 cm^−1^) is split into two components corresponding to CaCO_3_ and Na_2_CO_3_ [28,29,31]. This effect is manifested as a gradual shift of the main band from 1427 cm^−1^ for pure CaCO_3_ to 1449 cm^−1^ in LS50, accompanied by the formation of a shoulder in the 1420–1430 cm^−1^ region. The introduction of MgO into the LS series produces a similar broad band in the 500–400 cm^−1^ region.

**Figure 5:**
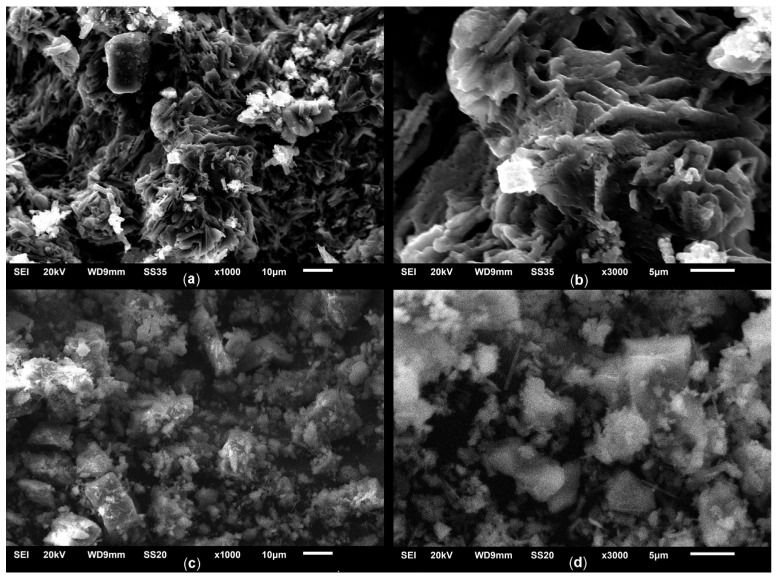
SEM micrographs of the KS25 (**a**,**b**) and LS25 (**c**,**d**) samples, at magnifications of 1000× (**a**,**c**) and 3000× (**b**,**d**).

The SEM micrographs of the KS25 and LS25 samples are shown in Figure 5.

The SEM analysis provided insights into the influence of support type on the morphology of the sorbents. In the case of the Na_2_CO_3_/kaolin sorbents (Figure 5a,b), a characteristic plate-like structure of kaolin is observed [32], consisting of loosely packed aggregates of ordered lamellae. At higher magnification (Figure 5b), finely dispersed inclusions are detected on the surface of the support, evenly distributed and likely corresponding to carbonate-containing phases.

In the case of CaCO_3_-based sorbents (Figure 5c,d), the surface structure appears more amorphous, which is typical of natural limestone morphology. At a magnification of 3000× (Figure 5d), isometric crystallites of the active phase are visible, localized against the background of the amorphous support.

The results of the elemental composition analysis of the samples, obtained using energy-dispersive X-ray spectroscopy (EDS), are summarized in Table 1.

The elemental analysis data (Table 1) reveal distinct differences in the distribution of the active component (Na) between the KS and LS series. For the KS series, the Na concentration remains approximately constant as the Na_2_CO_3_ loading increases, but it is consistently higher than the nominal loading value. In contrast, the LS series exhibits a proportional increase in Na content with the increase in Na_2_CO_3_ loading in the sorbents.

This discrepancy is likely due to differences in the distribution mechanisms of the active phase. In the Na_2_CO_3_/kaolin sorbents, Na is uniformly distributed on the surface of the support, forming a thin yet continuous surface phase, which is readily detectable by SEM (analysis depth of 1–2 μm). Conversely, in the Na_2_CO_3_/CaCO_3_ sorbents, the active phase crystallizes within the bulk of the support, forming discrete crystallites. As a result, the Na content determined by SEM in the LS series closely correlates with the actual Na_2_CO_3_ loading in the sample.

**Figure 6:**
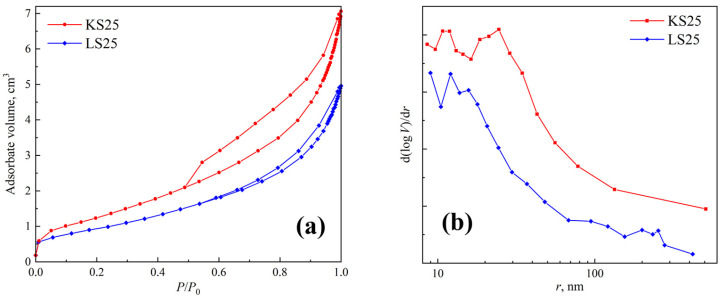
Characteristics of the pore structure of KS25 and LS25 sorbent samples: (**a**) BET nitrogen adsorption isotherms and (**b**) pore size distributions.

The pore structure of the sorbents was characterized using the BET method. The results of texture and adsorption characteristics measurements are summarized in Table 2. Nitrogen adsorption–desorption isotherms for the KS25 and LS25 samples are shown in Figure 6a. Based on these data, the pore size distributions were calculated and plotted as d(log V)/dr vs. log r (Figure 6b), providing insights into the pore structure characteristics and dominant pore sizes in the sorbents.

The obtained data indicate that an increase in Na_2_CO_3_ in the sorbents, both for kaolin-based and CaCO_3_-based samples, leads to a noticeable increase in porosity. This observation suggests that Na_2_CO_3_ partially forms its own pore structure during recrystallization in the course of synthesis. Furthermore, considering the XRD results, it can be inferred that the wet mixing and extrusion process contributes to the partial disaggregation of support particles, further enhancing the pore volume and specific surface area.

According to the BET adsorption–desorption isotherms and pore size distributions (Figure 6), the KS25 sorbent is characterized by a predominantly slit-shaped pore structure. This morphology is likely associated with the intrinsic plate-like structure of kaolin aggregates, with the active phase forming a surface film. The pore size distribution curve for the KS25 sample reveals a structure dominated by large micropores and mesopores, with two maxima at ~10 and 25 nm.

In contrast, the pore structure of the LS25 sample is defined by bottle-shaped pores with a less ordered geometry, formed between the particles of the support and the active phase, with a similar pore size distribution. The pore volume and specific surface area of LS25 are somewhat lower than those of KS25, which can be attributed to both the different mechanisms of active phase formation and the lower porosity of the initial CaCO_3_ compared to kaolin (specific surface area of 3.5 m^2^/g for CaCO_3_ vs. 12.6 m^2^/g for kaolin).

**Figure 7:**
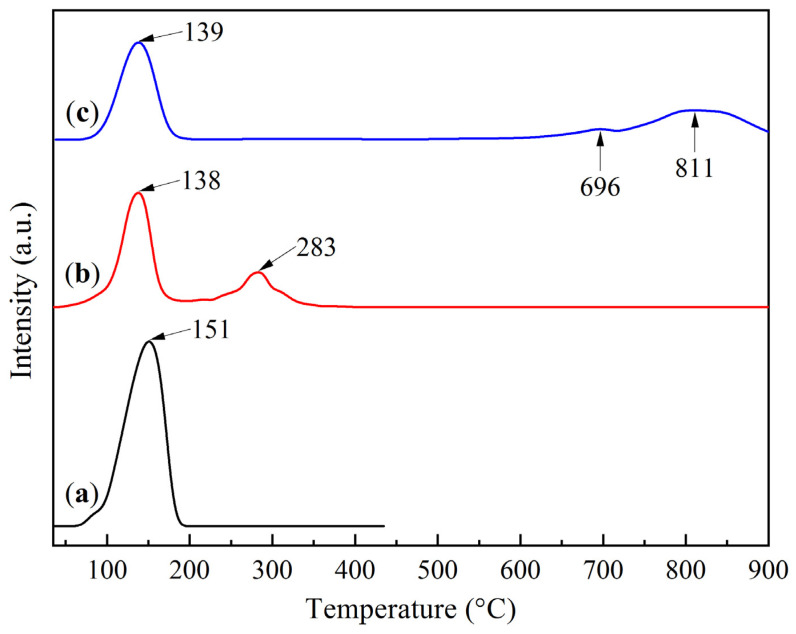
Temperature-programmed desorption profiles of samples: (**a**) NaHCO_3_, (**b**) KS25, and (**c**) LS25.

The activity of the sorbents was determined by CO_2_ temperature-programmed desorption (TPD) using pre-saturated samples. The influence of the support on the behavior of the active phase during thermal desorption was studied. Sodium bicarbonate (NaHCO_3_, 99.9%) was used as a standard. The results of the study in the temperature range of 20–900 °C are presented in Figure 7.

The TPD profiles of the KS25 and LS25 samples exhibit low-temperature desorption peaks, which are analogous to the desorption peak of sodium bicarbonate (NaHCO_3_), corresponding to its decomposition into sodium carbonate (Na_2_CO_3_).

2NaHCO_3_ → Na_2_CO_3_ + H_2_O + CO_2_. (1)

The desorption maxima for the samples with the active component are shifted to lower temperatures compared to pure NaHCO_3_. This shift can be attributed to the fine dispersion of the active phase over the support surface.

In addition to the low-temperature peaks, the KS25 sample shows a high-temperature peak at 283 °C, while the LS25 sample exhibits high-temperature peaks at 696 °C and 811 °C. These high-temperature peaks are likely associated with the decomposition of stable carbonate structures formed due to the interaction of the active phase with the functional groups of the support surface. For the KS25 sample, this may involve the formation of bridging bonds (Si–O–Na and Al–O–Na) resulting from the interaction of Na^+^ with hydroxyl groups of the kaolinite phase. In the case of LS25, the high-temperature peaks are attributed to the decomposition of the support phase with CaO formation.

The kinetic analysis of the CO_2_ desorption stages revealed that the activation energies for the low-temperature peaks, corresponding to NaHCO_3_ decomposition, are in the range of 65–70 kJ/mol, which is consistent with the literature data [33]. In contrast, the activation energies for the high-temperature peaks are significantly higher: 75 kJ/mol for KS25 and 116 kJ/mol for LS25. These higher values indicate the enhanced thermal stability of the respective phases.

**Figure 8:**
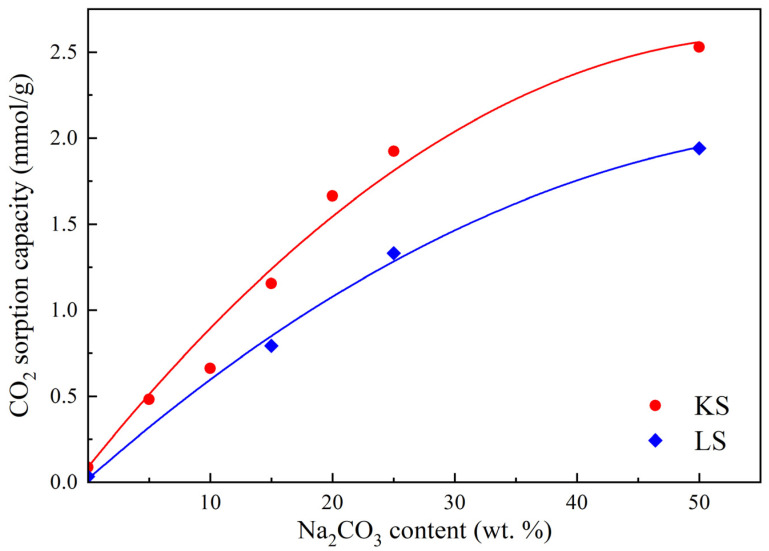
The influence of the active component content on the sorption capacity of sorbent samples.

The CO_2_ sorption capacities of the KS25 and LS25 series samples with varying active component loading are presented in Table 2 and Figure 8.

The sorption capacity values of the KS25 and LS25 series samples (Table 2, Figure 8) are consistent with the literature data for carbonate-based sorbents (1.8 mmol/g for 36.8% K_2_CO_3_/Al_2_O_3_ [16], 1.4 mmol/g for 30% Na_2_CO_3_/Al_2_O_3_) [20]. The results demonstrate a monotonic increase in sorption capacity with an increase in active phase loading in both series of sorbents. This trend indicates that CO_2_ has uninhibited access to the Na_2_CO_3_ surface, as well as the lack of strong interaction between the active phase and the support within the studied loading range.

However, for the samples with the highest Na_2_CO_3_ loading (KS50 and LS50), a deviation from the linear correlation of sorption capacity with Na_2_CO_3_ loading is observed. This deviation can be attributed to two main factors: the formation of new phases, such as thermonatrite (Na_2_CO_3_·H_2_O) in KS50, which may alter the adsorption behavior, as well as partial pore blockage due to the active component excess, reducing the accessible surface area for CO_2_ chemisorption. The observed decrease in specific activity, along with a sharp decline in formability, characterized by the number of fragmented granules during forming and drying, suggests that an optimal Na_2_CO_3_ loading of 25 wt.% is most suitable for both types of sorbents, providing a balance between efficiency and material cost.

### 2.2. Thermal Stability and Cyclic Durability of the Supported Sorbents

In the development of carbonate-based sorbents for CO_2_ capture, particularly within the context of Ca-loops and the dry carbonate process, special attention is given to their stability under high-temperature conditions [10,11,12]. Thermal stability, determined as the material’s ability to maintain its structure and activity under both thermal shocks and cyclic heating conditions, is a critical parameter for CaO-based systems, which typically operate at temperatures of 800–950 °C [10,34,35]. However, it is also crucial for sorbents used in the DCP: it is known that the CO_2_ adsorption rate increases with the desorption temperature up to 300 °C [16]. This necessitates the use of materials that are either inherently thermally stable or capable of regeneration during practical operation. Despite the importance of this property, systematic studies on the thermal stability of such adsorbents remain limited, primarily focusing on alumina, activated carbon, and certain metal oxides [17,18].

**Figure 9:**
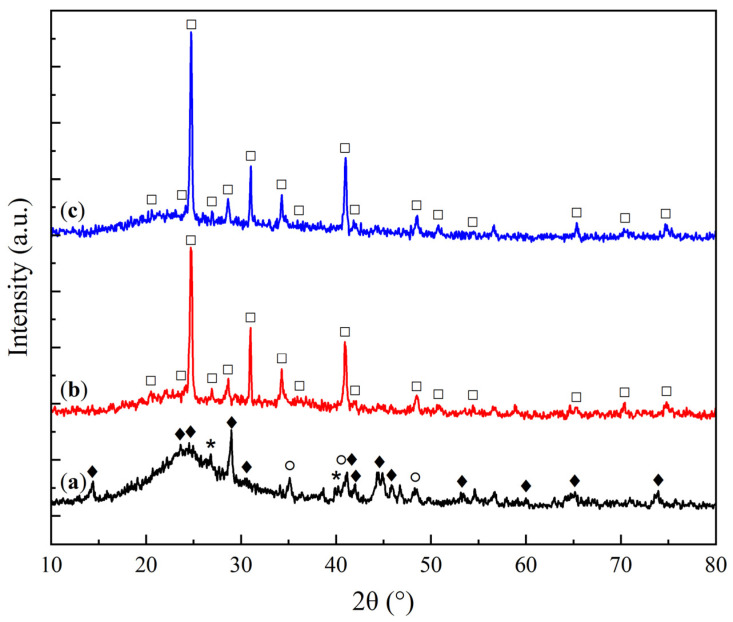
XRD patterns of the sorbent samples: (**a**) KS25, (**b**) KS25(750), and (**c**) KS25(750+H_2_O). (♦—kaolinite Al_2_Si_2_O_5_(OH)_4_, *—nahcolite NaHCO_3_, ○—natrite Na_2_CO_3_, □—carnegieite NaAlSiO_4_.)

**Figure 10:**
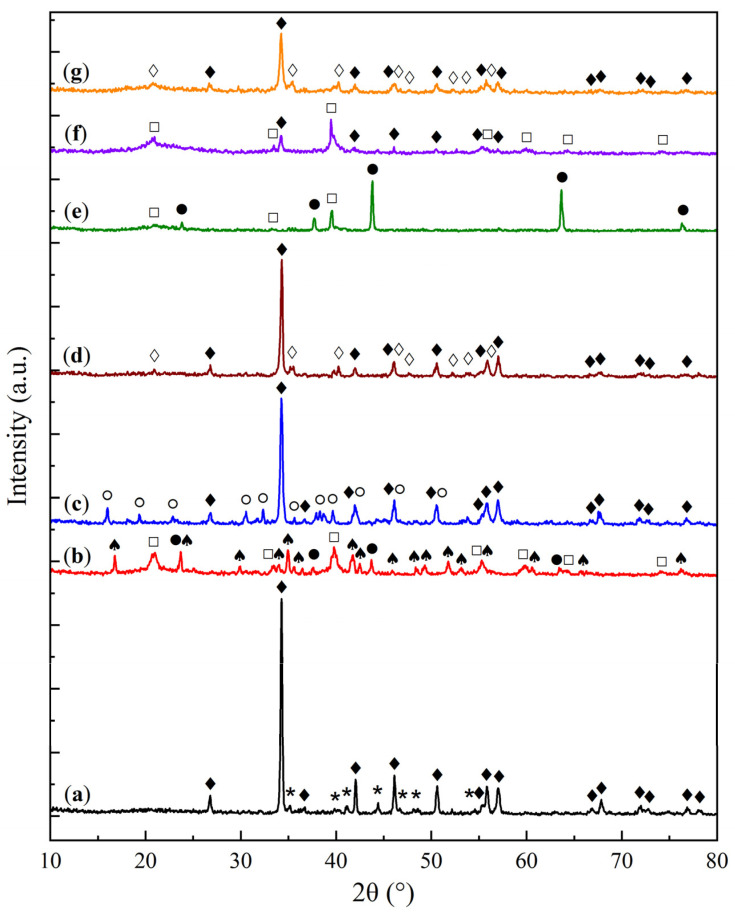
XRD patterns of the sorbent samples: (**a**) LS25, (**b**) LS25(750), (**c**) LS25(750+H_2_O), (**d**) LS25(750+H_2_O+CO_2_), (**e**) LS25(1000), (**f**) LS25(1000+H_2_O), and (**g**) LS25(1000+H_2_O+CO_2_). (♦—calcite CaCO_3_, *—natrite Na_2_CO_3_, ●—lime CaO, □—portlandite Ca(OH)_2_, ♠—nyerereite Na_2_Ca(CO_3_)_2_, ○—gaylussite Na_2_Ca(CO_3_)_2_·5H_2_O, ◊—nahcolite NaHCO_3_.)

To evaluate the thermal stability and regeneration capacity of the optimal sorbent samples (KS25 and LS25), the materials were subjected to high-temperature thermal treatment followed by regeneration in a humid environment and subsequent re-saturation in a humid CO_2_ flow. The impact of each stage was assessed using XRD and FTIR methods. The XRD results for the KS25 and LS25 sorbents after each treatment stage are presented in Figure 9 and Figure 10, respectively.

According to the XRD data (Figure 9 and Figure 10), when subjected to thermal treatment at 750 °C, the KS25 and LS25 sorbents undergo fundamentally different phase transformations. For the KS25 sample (Figure 9b), the calcination process leads to the disappearance of the characteristic reflections of kaolinite, NaHCO_3_, and Na_2_CO_3_ phases, accompanied by the formation of carnegieite (NaAlSiO_3_, JCPDS 11-220 [36]). This phase presumably forms as a result of the high-temperature interaction between kaolinite and Na_2_CO_3_, according to the following reaction:

Na_2_CO_3_ + Al_2_Si_2_O_5_(OH)_4_ → 2NaAlSiO_4_ + CO_2_ + 2H_2_O. (2)

The calculated elemental ratio of Na:Al:Si in the calcined sample is approximately 1:1:1, which is consistent with the formation of NaAlSiO_4_ according to this reaction. Subsequent regeneration of the calcined KS25 sorbent (Figure 9c) did not cause the restoration of the initial phases, indicating that the phase transition is irreversible under the given thermal conditions.

In the case of the LS25 sorbent (Figure 10b), calcination at 750 °C leads to the disappearance of CaCO_3_ and Na_2_CO_3_ diffraction maxima and the appearance of peaks corresponding to CaO (JCPDS 37-1497 [25]), Ca(OH)_2_ (JCPDS 44-1481 [25]), and a mixed carbonate phase—nyerereite (Na_2_Ca(CO_3_)_2_, JCPDS 33-1221 [37]). The formation of nyerereite is likely the result of a reaction between the support and the active component:

Na_2_CO_3_ + CaCO_3_ → Na_2_Ca(CO_3_)_2_. (3)

The presence of an excess of CaCO_3_ in the initial sorbent explains the simultaneous formation of the mixed salt and the CaO and Ca(OH)_2_ phases. The latter phase likely forms due to the interaction of CaO with atmospheric moisture.

Regeneration of the LS25(750) sample in a humid environment (Figure 10c) resulted in the formation of CaCO_3_ and the hydrated double salt—gaylussite (Na_2_Ca(CO_3_)_2_·5H_2_O, JCPDS 21-343 [38]). The formation of CaCO_3_ is attributed to the reaction of CaO and Ca(OH)_2_ with the CO_2_ present in the atmosphere [25], while gaylussite is formed through the hydration of nyerereite [37,38].

Re-saturation of the regenerated sample (Figure 10d) led to the decomposition of the double salt with the formation of CaCO_3_ and NaHCO_3_, likely according to the following reaction:

Na_2_Ca(CO_3_)_2_·5H_2_O + CO_2_ → 2NaHCO_3_ + CaCO_3_ + 4H_2_O. (4)

To further assess the thermal stability and regeneration capacity of the Na_2_CO_3_/CaCO_3_ system under severe conditions, the LS25 sample was subjected to thermal treatment at 1000 °C—a temperature exceeding both the melting point of Na_2_CO_3_ and the decomposition temperature of CaCO_3_ [39]. The XRD pattern of the LS25(1000) sample (Figure 10e) shows the presence of CaO and Ca(OH)_2_ phases, while no reflections corresponding to nyerereite are observed. This result is consistent with the literature data indicating the instability of this double salt under such conditions [39]. The absence of diffraction maxima of sodium carbonates is likely due to the amorphization of Na_2_CO_3_ upon melting. Despite their complete amorphization at 1000 °C, Na-containing phases are capable of partial regeneration, presumably due to the recrystallization of the support phase in these conditions.

Following regeneration of this sample in a humid environment (Figure 10f), Ca(OH)_2_ and CaCO_3_ phases are detected, but still no Na-containing phases are present. This observation suggests that the active phase remains in an amorphous state. Subsequent carbonation (Figure 10g) leads to the formation of NaHCO_3_ and CaCO_3_ phases, similar to the behavior observed for LS25(750+H_2_O+CO_2_). These results confirm the ability of the Na_2_CO_3_/CaCO_3_ system to restore its sorption capacity even after thermal treatment at 1000 °C.

**Figure 11:**
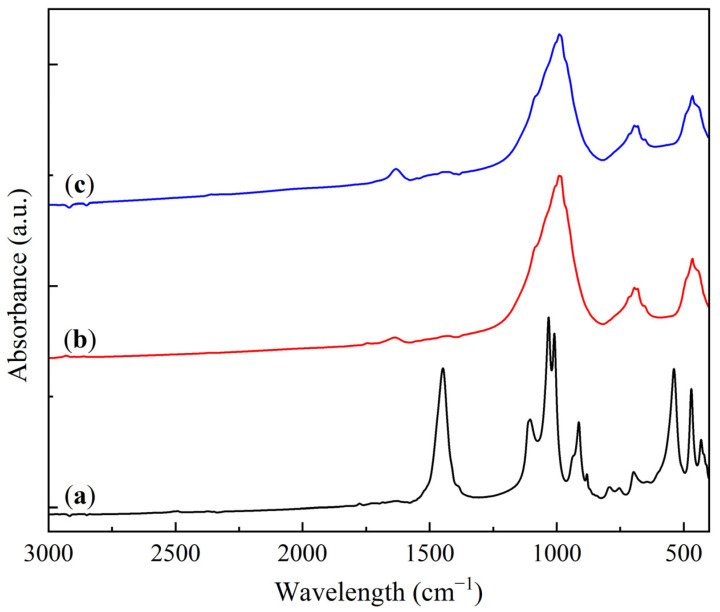
FTIR spectra of the sorbent samples: (**a**) KS25, (**b**) KS25(750), and (**c**) KS25(750+H_2_O).

**Figure 12:**
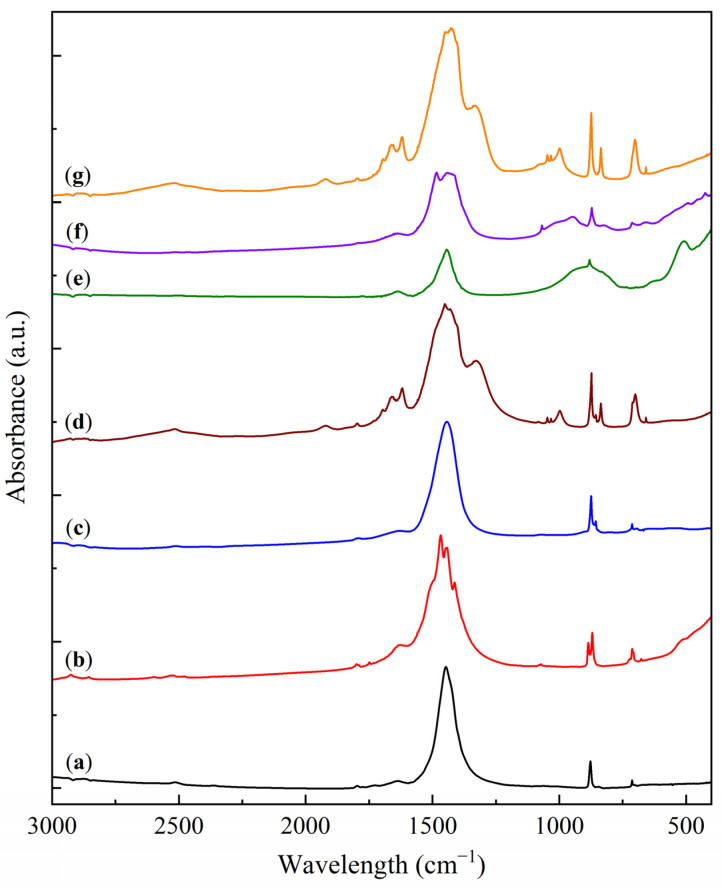
FTIR spectra of the sorbent samples: (**a**) LS25, (**b**) LS25(750), (**c**) LS25(750+H_2_O), (**d**) LS25(750+H_2_O+CO_2_), (**e**) LS25(1000), (**f**) LS25(1000+H_2_O), and (**g**) LS25(1000+H_2_O+CO_2_).

The results of the FTIR study of the heat treatment of the KS25 and LS25 samples are presented in Figure 11 and Figure 12.

According to the FTIR data, thermal treatment of the KS25 sorbent at 750 °C (Figure 11b) leads to the disappearance of the characteristic absorption bands of kaolinite in the region of 1100–400 cm^−1^. Instead, three broad bands appear at 982, 694, and 465 cm^−1^, corresponding to the spectra of framework aluminosilicates such as nepheline or carnegieite [40], in agreement with the XRD data. Upon regeneration of the sample (Figure 11c), the FTIR spectrum remains unchanged, confirming the irreversible phase transition of kaolinite under thermal treatment at 750 °C.

In the case of LS25, thermal treatment at 750 °C (Figure 12b) results in the splitting of the broad band at 1400–1500 cm^−1^ into four distinct peaks (1507, 1467, 1443, and 1413 cm^−1^), along with the appearance of new bands at 886, 870, and 712 cm^−1^. These absorption bands are characteristic of nyerereite [41], while a broad halo at 400–550 cm^−1^ indicates the presence of CaO [30].

After regeneration of the LS25 sample (Figure 12c), the splitting effect in the 1400–1500 cm^−1^ region disappears, and the spectrum is dominated by absorption bands at 1447, 874, 856, and 713 cm^−1^, characteristic of carbonate groups in gaylussite [42]. Subsequent carbonation (Figure 12d) results in the appearance of bands corresponding to both CaCO_3_ (713, 875, 1428 cm^−1^) and NaHCO_3_ (700, 835, 995, 1032, 1047, 1328, 1607, 1661, 1923 cm^−1^) [28,29]. The resulting spectrum is a superposition of the characteristic bands of CO_3_^2−^ and HCO_3_^−^ groups, corresponding to CaCO_3_ and NaHCO_3_ phases, respectively.

Thermal treatment of LS25 at 1000 °C (Figure 12e) leads to the retention of weak carbonate bands (1444 and 880 cm^−1^), along with the appearance of broad absorption regions (400–500 cm^−1^) characteristic of CaO [30]. This indicates the decomposition of CaCO_3_ to CaO, while Na_2_CO_3_ melts and transitions to an amorphous state without decomposition. After regeneration in a humid medium (Figure 12f), the CaO halo at 400–500 cm^−1^ disappears, while the intensity of the carbonate bands increases, indicating the hydration of CaO to Ca(OH)_2_, followed by partial carbonation in air. The spectrum of the LS25(1000+H_2_O+CO_2_) sample (Figure 12g) is similar to that of LS25(750+H_2_O+CO_2_), confirming the regeneration capability of the system after high-temperature treatment.

The sorption capacity values of the LS25 samples at different stages of thermal treatment and regeneration are summarized in Table 3.

Thermal treatment of the KS25 sample at 750 °C resulted in a 93.8% reduction in CO_2_ sorption capacity compared to the initial value. Regeneration failed to restore the capacity value, indicating irreversible deactivation of the sorbent. This deactivation is likely due to the formation of an inactive carnegieite phase and sintering of the pore structure.

In contrast, the LS25 sorbent demonstrated significantly better thermal stability. After thermal treatment at 750 °C followed by regeneration, the sorption capacity decreased by 6.6%. This capacity retention can be attributed to the recovery of carbonate phases and the formation of gaylussite, which exhibits adsorption activity, unlike the inactive nyerereite phase. After subjecting the LS25 sample to thermal treatment at 1000 °C followed by regeneration, the sorption capacity decreased by 31.2%, indicating a decline in regeneration efficiency at temperatures above the melting point of Na_2_CO_3_ and the decomposition temperature of the support.

**Figure 13:**
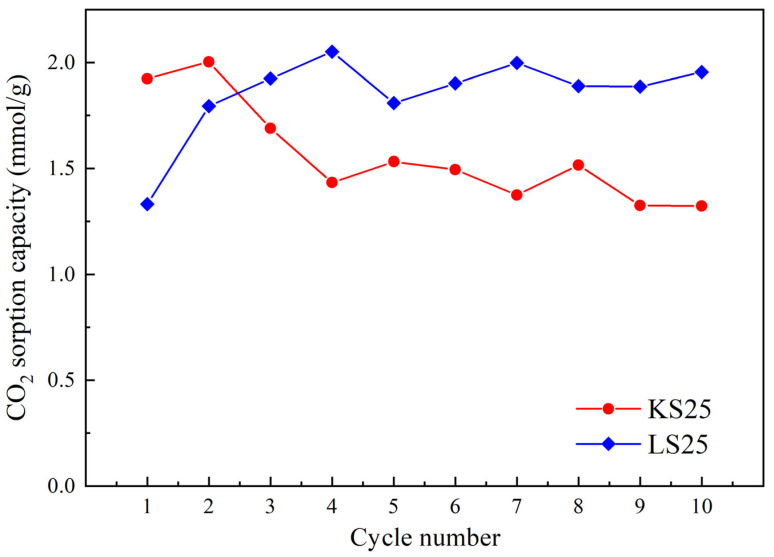
CO_2_ sorption capacity of KS25 and LS25 sorbents in adsorption–regeneration cycles.

To further assess the thermal stability of the sorbents, cyclic adsorption–desorption tests were performed on the KS25 and LS25 samples. The results are presented in Figure 13.

As shown in Figure 13, the KS25 sample exhibits a decrease in CO_2_ sorption capacity during the first four adsorption–desorption cycles, after which the capacity stabilizes at a constant level. In contrast, the LS25 sorbent shows an initial increase in sorption capacity during the first few cycles, followed by stabilization at a level higher than the initial value. This increase can be explained by several possible mechanisms, including partial removal of residual water and recrystallization of the amorphous Na_2_CO_3_ phase during the first cycles, contributing to an increase in the available surface; the formation of nyerereite and then gaylussite through the interaction between the components during the thermal cycles; and partial destruction of agglomerates of the active phase, improving wettability and the contact surface between CO_2_, H_2_O and Na_2_CO_3_. These results confirm the superior durability of the CaCO_3_-based sorbent under repeated adsorption–desorption cycles. This quality, combined with high thermal stability and regeneration capability, makes LS25 a promising candidate for practical applications in CO_2_ capture technologies.

### 2.3. Kinetic Study of CO_2_ Adsorption on the Na_2_CO_3_/CaCO_3_ Sorbent

Based on the results of the studies, the LS25 sorbent was selected for the investigation of CO_2_ chemisorption kinetics due to its superior stability.

Various models have been proposed to describe the kinetics of CO_2_ uptake by solid sorbents based on alkali metal carbonates, including first-order and second-order reaction equations with respect to CO_2_, as well as the Avrami–Erofeev equation [11,43,44,45]. Guo et al. [44] showed the applicability of a model that accounts for diffusion processes at the interface between the bicarbonate and carbonate phases—the reaction product and the initial active component—for the K_2_CO_3_/AC system. Quantum chemical calculations (DFT) of the Na_2_CO_3_ carbonation reaction [46,47] further confirmed this mechanism, which is based on the diffusion of CO_2_ and H_2_O (or H^+^ and HCO_3_^−^ ions) to the phase boundary between NaHCO_3_ and Na_2_CO_3_.

Taking this into account, the present study employed the approach proposed in [44] to analyze the carbonation kinetics of the LS25 sorbent, using the shrinking core model, which allows for the derivation of the following equation:

(5) $$t=\frac{R}{k\cdot p_{CO_{2}}^{0}p_{H_{2}O}^{0}}\left[1-{\left(1-f\left(t\right)\right)}^{\frac{1}{3}}\right]\;,$$

where *t* is the current time of the carbonation reaction (min), *R* is the average radius of the sorbent particles (mm), *k* is the carbonation rate constant (cm/min), $p_{CO_{2}}^{0},p_{H_{2}O}^{0}$ are the partial pressures of the initial components (CO_2_ and H_2_O), atm, and *f*(*t*) = $Q_{CO_{2}}/n_{CO_{2}}$ is the carbonation degree, defined as the ratio of the amount of CO_2_ adsorbed to the theoretical amount of CO_2_ that can be adsorbed by the sorbent sample with the given Na_2_CO_3_ concentration.

**Figure 14:**
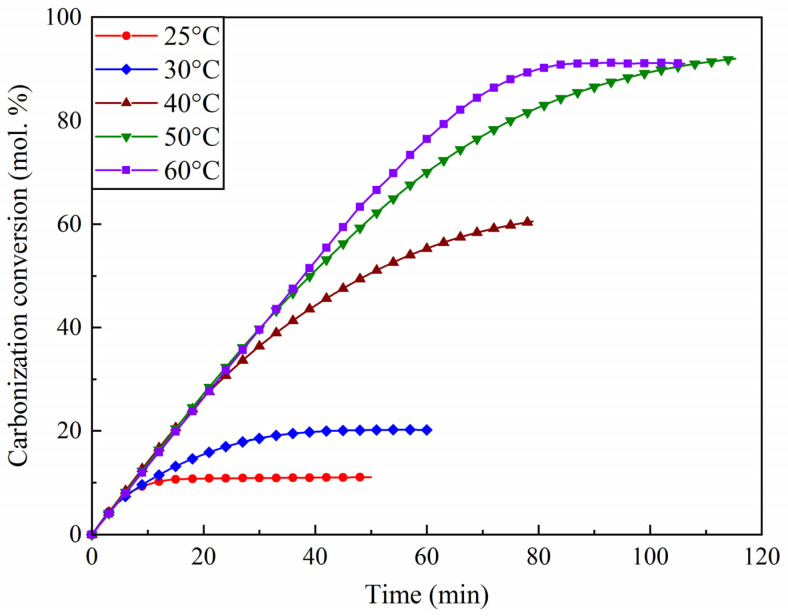
Kinetics of CO_2_ adsorption on the LS25 sorbent sample at different adsorption temperatures.

The kinetic study involved measuring the amount of CO_2_ captured by the sorbent over time under fixed conditions (temperature and gas composition). The resulting kinetic curves, representing the change in the carbonation degree as a function of time at various sorption temperatures, are shown in Figure 14.

Contrary to the results reported for the K_2_CO_3_/AC system [44], where an increase in adsorption temperature led to a decrease in sorption efficiency, the LS25 sorbent demonstrated a different behavior, with an increase in adsorption temperature resulting in higher final carbonation degrees and prolonged effective sorbent operation time. This discrepancy is likely due to the difference in the nature of the support: in carbon-based sorbents (K_2_CO_3_/AC), increasing the temperature reduces sorption efficiency due to the weakening of physical adsorption. In the Na_2_CO_3_/CaCO_3_ system, sorption is likely of a mostly chemical nature, as suggested by the low values of specific surface area and pore volume of the sorbent.

**Figure 15:**
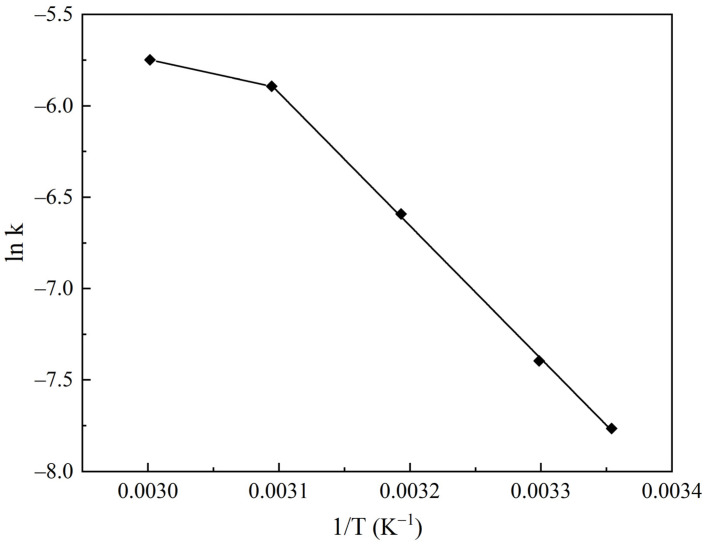
Arrhenius plot of the adsorption rate constant on the LS25 sorbent sample.

The carbonation curves (Figure 14) were processed using the shrinking core model. The calculated values of the adsorption rate constants are presented in Table 4, and their temperature dependence is illustrated in the Arrhenius plot (Figure 15).

In the temperature range of 25–50 °C, the carbonation kinetics are well described by the shrinking core model, with an activation energy of 60 kJ/mol. This value indicates that the process is not limited by diffusion constraints but rather by a chemical reaction at the phase boundary between NaHCO_3_ and Na_2_CO_3_. At higher temperatures, the rate increase slows down, which can be attributed to diffusion limitations: as the adsorption temperature is raised, the rate-determining step shifts from chemical reaction (chemisorption) to mass transfer in the pore structure of the sorbent, presumably defined by Knudsen diffusion [48,49]. These findings confirm the high efficiency of the LS25 sorbent for low-temperature CO_2_ capture.

## 3. Materials and Methods

### 3.1. Synthesis of Sorbent Samples

The sorbent samples were synthesized by mixing dry powders of the support material (kaolin or calcium carbonate) and the active component (Na_2_CO_3_). The resulting dry mixture was moistened with water until a mass suitable for molding was obtained (moisture content 20%). The sorbents were granulated using an extrusion method with a screw extruder equipped with 3 mm diameter dies. The resulting extrudates were cured overnight and then dried at 120 °C for 4 h.

The sorbents were prepared with active component loadings up to 50 wt.% Na_2_CO_3_. Kaolin-based samples were designated as KS, while those based on CaCO_3_ were labeled as LS, with the percentage of Na_2_CO_3_ specified in the sample code (e.g., KS25 for 25 wt.% Na_2_CO_3_). In addition, to enhance mechanical stability and overall performance, some samples were modified with magnesia (MgO) at 10 wt.% of the support mass, which is reported to improve the durability of carbonate-containing materials [50,51]. These modified samples were labeled KS25M and LS25M, respectively.

For sorption capacity determination, the sorbent samples were pre-saturated with CO_2_. Saturation was carried out in a flow quartz reactor with a humid gas mixture (2% CO_2_, 5% H_2_O, balance He) for 8 h at room temperature.

To assess thermal stability, the KS25 and LS25 samples were subjected to high-temperature treatment, followed by regeneration and re-saturation with CO_2_. Samples calcined at a specific temperature (T, °C) were labeled as KS25(T) and LS25(T). Samples calcined and regenerated in a humid environment (5% H_2_O, balance He) were labeled as KS25(T+H_2_O) and LS25(T+H_2_O). Samples that underwent further re-saturation in a wet CO_2_ stream (2% CO_2_, 5% H_2_O, balance He) were labeled as KS25(T+H_2_O+CO_2_) and LS25(T+H_2_O+CO_2_), respectively.

### 3.2. Characterization of Sorbent Samples

The physicochemical characteristics of the sorbent samples were investigated using a range of analytical techniques. X-ray diffraction (XRD) patterns were obtained using a DRON-4-07 powder diffractometer (Burevestnik, Saint-Petersburg, Russia) with Co Kα radiation and a Ni filter, operating at the parameters of 30 kV and 20 mA. The scanning range was 10–80°, with a 0.02° step size and a 2°/min scanning speed. The average crystallite size was calculated as follows using the Scherrer equation [19]:

(6) $$d=\frac{0.9\lambda }{B\cos\theta }\;,$$

where *d* is the crystallite mean size (nm), λ = 0.1789 is the wavelength (nm), *B* is the full peak width at half maximum (rad), and θ is the diffraction angle (°).

The structure of the samples was studied using the SEM method with a low-vacuum scanning electron microscope JSM-6610LV (JEOL, Tokyo, Japan) equipped with an energy-dispersive X-ray spectroscopy (EDS) module for surface elemental analysis.

The FTIR study was conducted on a Nicolet iS5 FTIR spectrometer (Thermo Fisher Scientific, Milan, Italy) at room temperature in the range of 4000–400 cm^−1^. The samples were pressed into KBr pellets.

The specific surface area and pore characteristics of the samples were measured using a Thermo Scientific Surfer gas adsorption analyzer (Thermo Fisher Scientific, Italy) at 77 K with nitrogen (N_2_) used as the adsorbate, and the specific surface area was calculated by the Brunauer–Emmett–Teller (BET) method.

The CO_2_ sorption capacity of pre-saturated, crushed sorbent samples (0.5 g) was determined by TPD using a flow quartz reactor equipped with a Chromatek-Crystal 5000 gas chromatograph (Chromatek, Yoshkar-Ola, Russia). The desorption was performed in a He flow (30 cm^3^/min) at a linear heating rate of 10 °C/min up to 900 °C (up to 150 °C in the case of adsorption–desorption cycles). The desorbed CO_2_ was detected using a thermal conductivity detector (TCD). For the kinetic analysis of TPD data, the curves were integrated and then processed in accordance with the 1st order equation, considering a constant rate of temperature increase [52].

The kinetics of CO_2_ adsorption were studied using pre-saturated, crushed sorbent samples: after TPD up to 150 °C, the samples were rapidly cooled to the target temperature and maintained in a liquid-thermostatted reactor under a humid CO_2_ flow (2% CO_2_, 5% H_2_O, balance He) at a flow rate of 30 cm^3^/min.

## 4. Conclusions

The study investigated molded Na_2_CO_3_/support sorbents with kaolin and CaCO_3_ as supports (series KS and LS, respectively) with varying active component loadings, confirming their effectiveness as CO_2_ sorbents under the dry carbonate process conditions (adsorption at 50–80 °C, desorption at 100–150 °C). Physicochemical analysis of the sorbent samples (XRD, FTIR, SEM) revealed the presence of the phases of the support (kaolinite or calcite) and the carbonate and bicarbonate phases, whose intensity increased with the rising Na_2_CO_3_ loading. It was established that the morphology of the support significantly affects the distribution of the active phase. In kaolin-based sorbents, Na_2_CO_3_ forms a surface film on the plate-like structure of the support. In CaCO_3_-based sorbents, the active component predominantly crystallizes within the bulk. This difference is also confirmed to be reflected in the pore structure formation. The optimal Na_2_CO_3_ content in both series was determined to be 25 wt.%, providing the best balance between sorption capacity and technological properties.

The thermal stability and cyclic performance of the sorbents with optimal Na_2_CO_3_ content (KS25 and LS25) were thoroughly studied. It was found that the thermal treatment of KS25 at 750 °C led to the formation of an inactive carnegieite phase and an irreversible loss of sorption capacity (93.8% reduction). In contrast, LS25 retained its regenerative capacity due to the formation of nyerereite (Na_2_Ca(CO_3_)_2_) upon thermal treatment and gaylussite (Na_2_Ca(CO_3_)_2_·5H_2_O) upon subsequent hydration. The gaylussite was further converted into NaHCO_3_ and CaCO_3_ upon re-saturation in CO_2_ flow, enabling nearly complete regeneration of the active phase. Thermal treatment of LS25 at 1000 °C resulted in a partial loss of activity (31.2% reduction), attributed to the amorphization of Na_2_CO_3_ upon melting. However, the sorbent retained its ability to regenerate, likely due to the recrystallization of NaHCO_3_ from the amorphous phase. The CaCO_3_ support underwent decomposition to CaO, which, after hydration and carbonation, returned to its original CaCO_3_ state.

Cyclic adsorption–desorption tests demonstrated that LS25 exhibited high stability over multiple cycles without degradation, unlike KS25, which showed a decrease in sorption capacity after the first four cycles. These findings highlight the superior thermal stability, regeneration capability, and operational durability of CaCO_3_-based sorbents.

The kinetics of CO_2_ chemisorption on the LS25 sorbent were investigated using the shrinking core model. It was determined that, in the temperature range of 25–50 °C, the process is governed by chemical reaction at the phase boundary between NaHCO_3_ and Na_2_CO_3_, with an activation energy of 60 kJ/mol. The observed increase in adsorption efficiency with temperature confirms the chemical nature of the sorption process. At higher temperatures (60 °C), a diffusion-limited regime is manifested.

In summary, the results of this study demonstrate that supported alkali metal carbonate sorbents are highly promising materials for low-temperature dry CO_2_ capture from flue gases. Moreover, they offer significant potential as the basis for sorbent–catalyst systems for the conversion of captured CO_2_ into valuable chemical products within an integrated reaction scheme.

## Figures and Tables

**Table 1:** Elemental composition of the samples of the KS and LS series.

Sample	O	Al	Si	Na	Mg	Ca
KS5	49.93	8.15	9.23	32.69	—	—
KS10	48.85	8.23	7.90	35.02	—	—
KS15	49.78	9.62	9.58	31.02	—	—
KS20	49.24	7.80	7.80	35.16	—	—
KS25	53.06	5.62	4.92	36.40	—	—
KS50	51.01	7.02	6.57	35.40	—	—
KS25M	46.51	5.51	5.69	33.45	8.84	—
LS15	52.95	0.83	0.25	6.95	0.16	38.86
LS25	55.78	0.27	1.42	8.48	0.34	33.71
LS50	54.62	0.41	0.55	25.38	0.69	18.35
LS25M	52.99	0.33	0.43	10.03	9.01	27.21

**Table 2:** Texture and adsorption characteristics of synthesized sorbent samples.

Sample	Bulk Density, g/cm^3^	Surface Area, m^2^/g	Pore Volume, cm^3^/g	Adsorption Capacity, mmol/g
KS5	0.85	13.3	0.084	0.482
KS10	0.76	13.7	0.086	0.662
KS15	0.72	16.2	0.102	1.156
KS20	0.70	17.9	0.112	1.664
KS25	0.68	20.7	0.130	1.923
KS50	0.63	25.1	0.149	2.529
KS25M	0.74	18.1	0.108	1.282
LS15	0.94	8.4	0.038	0.792
LS25	0.90	11.7	0.065	1.331
LS50	0.87	13.5	0.076	1.941
LS25M	0.72	17.9	0.100	1.324

**Table 3:** Adsorption capacity of the KS25 and LS25 sorbent samples under different treatment conditions.

Treatment Conditions	CO_2_ Sorption Capacity, mmol/g	
	KS25	LS25
Initial	1.923	1.331
After thermal treatment at 750 °C	0.113	0.170
After regeneration	0.119	1.244
After thermal treatment at 1000 °C	—	0.147
After regeneration	—	0.916

**Table 4:** Rate constants of CO_2_ sorption on the LS25 sorbent sample at different temperatures.

Temperature, °C	25	30	40	50	60
k · 103, cm/min	0.424	0.614	1.370	2.757	3.186

## Data Availability

The data presented in this study are available upon request from the corresponding authors.

## Abbreviations

The following abbreviations are used in this manuscript:

CCUS	Carbon Capture, Utilization, and Storage
CCU	Carbon Capture and Utilization
CCS	Carbon Capture and Storage
DCP	Dry Carbonate Process
XRD	X-ray Diffraction
JCPDS	Joint Committee on Powder Diffraction Standards
SEM	Scanning Electron Microscopy
FTIR	Fourier-Transform Infrared Spectroscopy
EDS	Energy-Dispersive X-ray Spectroscopy
BET	Brunauer–Emmett–Teller Method
TPD	Temperature-Programmed Desorption
AC	Active Carbon
DFT	Density Functional Theory
TCD	Thermal Conductivity Detector
